# Syntaxin Binding Protein 1 Is Not Required for Allergic Inflammation via IgE-Mediated Mast Cell Activation

**DOI:** 10.1371/journal.pone.0058560

**Published:** 2013-03-06

**Authors:** Zhengli Wu, Adam J. MacNeil, Jason N. Berman, Tong-Jun Lin

**Affiliations:** Department of Microbiology and Immunology and Department of Pediatrics, Dalhousie University and IWK Health Centre, Halifax, Nova Scotia, Canada; UTHealth Medical School, United States of America

## Abstract

Mast cells play a central role in both innate and acquired immunity. When activated by IgE-dependent FcεRI cross-linking, mast cells rapidly initiate a signaling cascade and undergo an extensive release of their granule contents, including inflammatory mediators. Some SNARE (soluble N-ethylmaleimide-sensitive fusion factor attachment protein receptor) proteins and SM (Sec1/Munc18) family proteins are involved in mast cell degranulation. However, the function of syntaxin binding protein 1 (STXBP1), a member of SM family, in mast cell degranulation is currently unknown. In this study, we examined the role of STXBP1 in IgE-dependent mast cell activation. Liver-derived mast cells (LMCs) from wild-type and STXBP1-deficient mice were cultured *in vitro* for the study of mast cell maturation, degranulation, cytokine and chemokine production, as well as MAPK, IκB-NFκB, and NFAT signaling pathways. In addition, *in vivo* models of passive cutaneous anaphylaxis and late-phase IgE-dependent inflammation were conducted in mast cell deficient W^sh^ mice that had been reconstituted with wild-type or STXBP1-deficient mast cells. Our findings indicate that STXBP1 is not required for any of these important functional mechanisms in mast cells both *in vitro* and *in vivo*. Our results demonstrate that STXBP1 is dispensable during IgE-mediated mast cell activation and in IgE-dependent allergic inflammatory reactions.

## Introduction

Mast cells are derived from CD34+ hematopoietic progenitor cells, and play an important role in both innate and acquired immunity [1]. Mature mast cells express the stem cell factor (SCF) receptor, c-Kit (CD117), and the high affinity IgE receptor, FcεRI [2]. Upon the aggregation of FcεRI, induced by polyvalent antigen recognized by IgE bound to the FcεRI receptor, mast cells become activated, resulting in the activation of a series of signaling cascades [3]. Mast cell activation leads to degranulation of preformed mediators and the production of *de novo* synthesized mediators such as cytokines, chemokines and lipid mediators [3], [4], [5]. The release of preformed and newly synthesized mediators can cause profound inflammatory effects in allergic diseases [6]. Mast cell degranulation, like other intracellular trafficking processes, depends on the interaction of vesicular v-SNAREs (soluble N-ethylmaleimide-sensitive fusion factor attachment protein receptor) and target t-SNAREs to form a core complex that catalyses membrane fusion. The Sec1/Munc18 (SM) family is essential in intracellular trafficking through interaction with SNAREs [7]. This SM-SNARE interaction is involved in compound exocytosis that requires the fusion of docked secretory granules with the plasma membrane [8], [9]. In the case of mast cell degranulation, many proteins are involved, including SNARE proteins (such as syntaxin-3 [10], syntaxin-4 [11], SNAP-23 [11], [12], VAMP-2 [13], VAMP-7 [10], and VAMP-8 [11]), and SM family proteins (such as STXBP2, STXBP3) [9], among others.

The SM family includes at least seven mammalian members: syntaxin binding protein (STXBP)1, STXBP2, STXBP3, VPS33A, VPS33B, VPS45, and SLY1. The STXBPs are functionally homologous to yeast Sec1p and function at the plasma membrane where they bind to the closed conformation of syntaxin 1–4 [14]. STXBP1 can play different roles in exocytosis regulated by various cellular machineries [15]. STXBP1 acts, along with STXBP2, to support the function of wide range of syntaxins and brings syntaxin-1 to the plasma membrane by binding the closed conformation of the protein [16]. STXBP1 also mediates synaptic vesicle docking and priming through direct binding to SNARE complexes [17], [18], [19], [20], and leads to the subsequent calcium-mediated initiation of fusion [17], [21], [22], [23]. Apart from its regulatory roles in vesicle docking, priming, and fusion, STXBP1 has been shown to bind double-stranded DNA and localize to neuronal nuclei [19]. It was proposed as a putative shuttle protein between the cytoplasm and the nucleus in neurons [19]. STXBP1 was shown to regulate neurite outgrowth from neurons through regulating cone filopodia [24], and negatively regulates insulin secretion by stabilizing syntaxin-1A in a closed conformation during vesicle priming [25].

Mutations in the *STXBP1* gene have been shown to be associated with a wide spectrum of epileptic disorders and intellectual disabilities, including early infantile epileptic encephalopathy, as well as symptomatic generalized, partial, and non-syndromic epilepsy [26], [27], [28], [29], [30], [31]. STXBP1 and its interaction with syntaxin-1A have been well studied in neurons [32], [33]. STXBP1 is phosphorylated by PKC *in vitro* and *in vivo*, and is critical for vesical secretion [34], [35]. Mechanisms of mast cell degranulation share many similar features of vesical secretion as those present in neurons, such as PKC activation [36], and involvement of SNARE proteins (syntaxin-3 [10], syntaxin-4 [11], SNAP-23 [11], [12], VAMP-2 [13], VAMP-7 [10], and VAMP-8 [11]) and SM family proteins (STXBP2, STXBP3) [9]. However, a role of STXBP1 in mast cells has not been previously investigated. In this study, we used liver-derived mast cells (LMCs) from *STXBP1*-deficient mice to examine whether STXBP1 is required for mast cell function. Surprisingly, we found no significant functional discrepancy between wild-type and STXBP1-deficient mast cells both *in vivo* and *in vitro* suggesting that STXBP1 is dispensable for mast cell maturation and IgE-dependent mast cell functions, and may point to functional redundancy in mast cell STXBPs.

## Materials and Methods

### Animals

Heterozygous STXBP1 mice (STXBP1^+/−^) on a C57BL/6 background were purchased from Jackson Laboratory (http://www.jax.org/). To minimize the effects of the genetic backgrounds, all mice were obtained by heterozygous mouse mating and littermate controls were used for all experiments. The protocols were approved by the University Committee on Laboratory Animals, Dalhousie University, in accordance with the guidelines of the Canadian Council on Animal Care.

### Antibodies

Antibodies to phospho-JNK (Thr-183/Tyr-185), JNK, phospho-p38 MAPK (Thr-180/Tyr-182), phospho-p44/42 (ERK1/2), p44/42 MAPK, phospho-IκB-α (Ser 32), IκB-α, phospho-Akt (Ser 473), Akt, STXBP1, and PKG-1 were purchased from Cell Signaling Technology, Inc. (Beverly, MA). Antibodies to p38 MAPK and actin were purchased from Santa Cruz Biotechnology (Santa Cruz, CA). Antibody to syntaxin-1 was purchased from Sigma (St. Louis, MO). FITC-conjugated rat anti-mouse IgE (IgG1), FITC-rat IgG1 and FITC-conjugated rat anti-mouse CD117 (c-kit) were purchased from Cedarlane Laboratories (Burlington, ON, Canada).

### Mast Cell Culture and Activation

Mouse liver-derived mast cells (LMC) were cultured, as previously described [37]. Briefly, liver tissue was removed and placed in a sterile environment where it was ground to produce a single cell suspension in RPMI 1640 medium. Cells were collected, centrifuged at 500×g for 5 min at 4°C, and resuspended at a density of 0.5×10^6^ cells/ml in complete medium (RPMI 1640 medium containing 10% FBS, 10% WEHI-3B conditioned medium, 30 ng/ml stem cell factor, 50 units/ml each of penicillin and streptomycin, 50 µM 2-mercaptoethanol, and 200 nM prostaglandin E_2_). An aliquot of cells from each mouse was used for genotyping. Nonadherent cells were resuspended in complete medium twice per week and transferred to a fresh flask once per week. Mast cells were confirmed by toluidine blue staining and flow cytometry analysis for c-Kit and IgE receptor expression (FACSAria). Following 4 wks in culture, mast cell purity was >98%. LMCs were passively sensitized with IgE from TIB-141 cells (American Type Culture Collection), and then activated by stimulation with 10 ng/ml trinitrophenyl (TNP)-BSA (Biosearch Technologies, Inc., Novato, CA) or 1 µM calcium ionophore A23187 (Sigma). Mast cell degranulation was determined by measuring β-hexosaminidase release.

### Reverse Transcription PCR (RT-PCR)

Total RNA was isolated using TRIzol reagent (Invitrogen) and reverse transcribed using SuperScript II Rnase H-Reverse Transcriptase (Invitrogen) according to the manufacturer’s instructions. cDNA was used to amplify members of Sec/Munc (SM) family [14] with the following primers: STXBP1 forward, 5′-CCC GAG CAG CCA AAG TCA TC-3′, and reverse, 5′-GGT CTT CTC GCC AGT GTT CA-3′, 605 bp; STXBP2 forward, 5′-CAC TAT TAC ACG AAC TCA CG-3′, and reverse, 5′-ATG AGG CTG CTG TAC GAC T-3′, 589 bp; STXBP3 forward, 5′-GGA AAA GAG AAG GAG GCA G-3′, and reverse, 5′-CCA AGG TGG CTC CAG TTA C-3′, 515 bp; VPS33A forward, 5′-CGA GTT CCT GGA CAA GTG C-3′, and reverse, 5′-TGA GTT CTG GCT TCC TGT GA-3′, 586 bp; VPS33B forward, 5′-AGC TGG CTG GCA GAA TTA G-3′, and reverse, 5′-ATT GCG GAT CTC ACT GAA C-3′, 566 bp; VPS45 forward, 5′-CTG AAC TTT GCC GAG ATT G-3′, and reverse, 5′-CTA ACC CGT TTA CCA CCA-3′, 473 bp; SLY1 forward, 5′-GGA TTC TGG GAA GAG TGG C-3′, and reverse, 5′-CAC AGG TGG CTT TTC AAC G-3′, 442 bp; and actin forward, 5′-ATC TGG CAC CAC ACC TTC TAC-3′, and reverse, 5′-GGA TGT CAA CGT CAC ACT TC-3′, 612 bp. Each pair of primers spans at least one intron on genomic localization.

### Enzyme-linked Immunosorbent Assay (ELISA) and Cytokine Multiplex Assay

Cytokines in the cell-free supernatants were determined using DuoSet ELISA kits obtained from R&D Systems (Minneapolis, MN). Prostaglandin D2 (PGD2) enzyme immunoassay kit was obtained from Cayman (Ann Arbor, MI). Cell-free culture supernatants were also analyzed for cytokines using a Bio-Plex Pro mouse23-plex group 1 cytokine assay (Bio-Rad, Hercules, CA), which detects 23 cytokines and chemokines. The assay was performed according to the manufacturer’s protocol and was read on a Bio-Plex 200 HTF multiplex array system. Data were analyzed using Bio-Plex Manager version 6.0 software (Bio-Rad).

### Western Blotting

Cells were lysed in radioimmunoprecipitation (RIPA) assay buffer supplemented with a panel of protease and phosphatase inhibitors. Cleared lysates (35 µg protein) were separated on 10% SDS-PAGE gels and transferred onto polyvinylidenedifluoride (PVDF) membrane. After blocking with 5% nonfat dry milk for 2 hours at room temperature, membranes were incubated with primary antibodies overnight at 4°C and secondary antibodies were incubated for 1 hour at room temperature, and finally detected via an ECL-detection system (Western Lightning Plus-ECL; PerkinElmer) on BioMax film (Kodak).

### Intracellular Calcium

Mast cells were incubated for 30 min with 2 µM Fura-2 AM (Invitrogen). After washing, mast cells were resuspended in phosphate buffer with 1.5 mM CaCl_2_ at a concentration of 1×10^6^ cells/ml. Fluorescence was measured by placing 2 ml of the mast cell suspension in a 37°C thermostated quartz cuvette with magnetic stirring in an RF-1501 spectrofluorophotometer (Shimadzu Co., Tokyo, Japan).

### Reactive Oxygen Intermediates (ROI)

To measure reactive oxygen intermediates (ROI), a fluorescence cytometry assay using intracellular oxidation of 2,7-dichlorofluoroscein diacetate (DCFH-DA, Sigma) was performed. IgE-sensitized mast cells were incubated with DCFH-DA at a final concentration of 5 µM for 15 min at 37°C and then stimulated with 10 ng/ml TNP-BSA for various times. After stimulation, cells were incubated on ice immediately, and then washed twice and resuspended in PBS. Cells were analyzed on a fluorescence-activated cell sorting (FACS) Calibur (BD Biosciences).

### Nuclear Extract Preparation and Electrophoretic Mobility Shift Assay (EMSA)

Nuclear protein extracts were obtained using a nuclear extract kit (Active Motif, Carlsbad, CA), according to the manufacturer’s protocol. All preparation procedures were carried out at 4°C. Total protein concentration was determined using the Bio-Rad protein assay (Bio-Rad Laboratories, Hercules, CA). EMSA was carried out as previously described [38]. The following double-stranded oligonucleotide probes were used: Egr binding consensus sequences 5′-GGA TCC AGC GGG GGC GAG CGG GGG CGA ACG-3′ (Geneka Biotechnology, Montreal, QC, Canada); NFκB binding consensus sequences on the mouse IL-6 promoter 5′-TTA TCA AAT GTG GGA TTT TCC CAT-3′; and NFAT binding consensus sequence on the mouse IL-13 promoter 5′-AAG GTG TTT CCC CAA GCC TTT CCC-3′. Samples (8 µg) were electrophoresed on native polyacrylamide gels with radiolabeled (^32^P) dsDNA probes, dried on filter paper for 2 h at 80°C, and exposed to BioMax Film (Kodak) at −80°C.

### IgE-mediated Passive Cutaneous Anaphylaxis and Late-phase Cutaneous Reactions

Mast cell-deficient W^sh^/W^sh^ (W^sh^) mice were reconstituted locally in the ears and hind paws by intradermal injection of wild-type (left ear and left hind paw) and STXBP1-deficient (right ear and right hind paw) mast cells. Eight weeks later, models of IgE-mediated passive cutaneous anaphylaxis and late-phase cutaneous reactions were evaluated. For IgE-mediated passive cutaneous anaphylaxis, W^sh^ mice with or without (background control) mast cell-reconstitution were sensitized by intradermal injection of 20 ng anti-DNP IgE mAb (Sigma-Aldrich) into each ear. After 24 h, mice were challenged by i.v. injection of 100 µg DNP-BSA in 200 µl of Evan’s blue dye (1% wt/vol; Sigma-Aldrich). Thirty min later, whole ears were collected in 300 µl of formamide and incubated at 80°C for 2 h in a water bath to extract the Evan’s blue dye. The absorbance was determined at 620 nm.

For IgE-mediated late-phase cutaneous reactions, mast cell reconstituted-W^sh^ mice were passively sensitized by i.v. injection of 2 µg anti-DNP IgE mAb (Sigma-Aldrich). After 24 h, a cutaneous reaction was elicited by the application of 20 µl DNFB (0.3% wt/vol; Sigma-Aldrich) in acetone/olive oil (4∶1) to both sides of the hind paw or ear. The thickness of the foot pad or ear was measured using a digital micrometer before and after DNFB treatment 24 h. The thicknesses of the ear or hind paw before DNFB treatment were used as the baseline value. The DNFB-induced increment of tissue thickness was expressed as a percentage of the baseline values.

### Statistical Analysis

The paired Student's *t* test was used for statistical evaluation of data. Results were considered significant when p<0.05. Data are expressed as means ± SEM.

## Results

### STXBP1 Deficiency does not Impair Mast Cell Maturation

**Figure 1:**
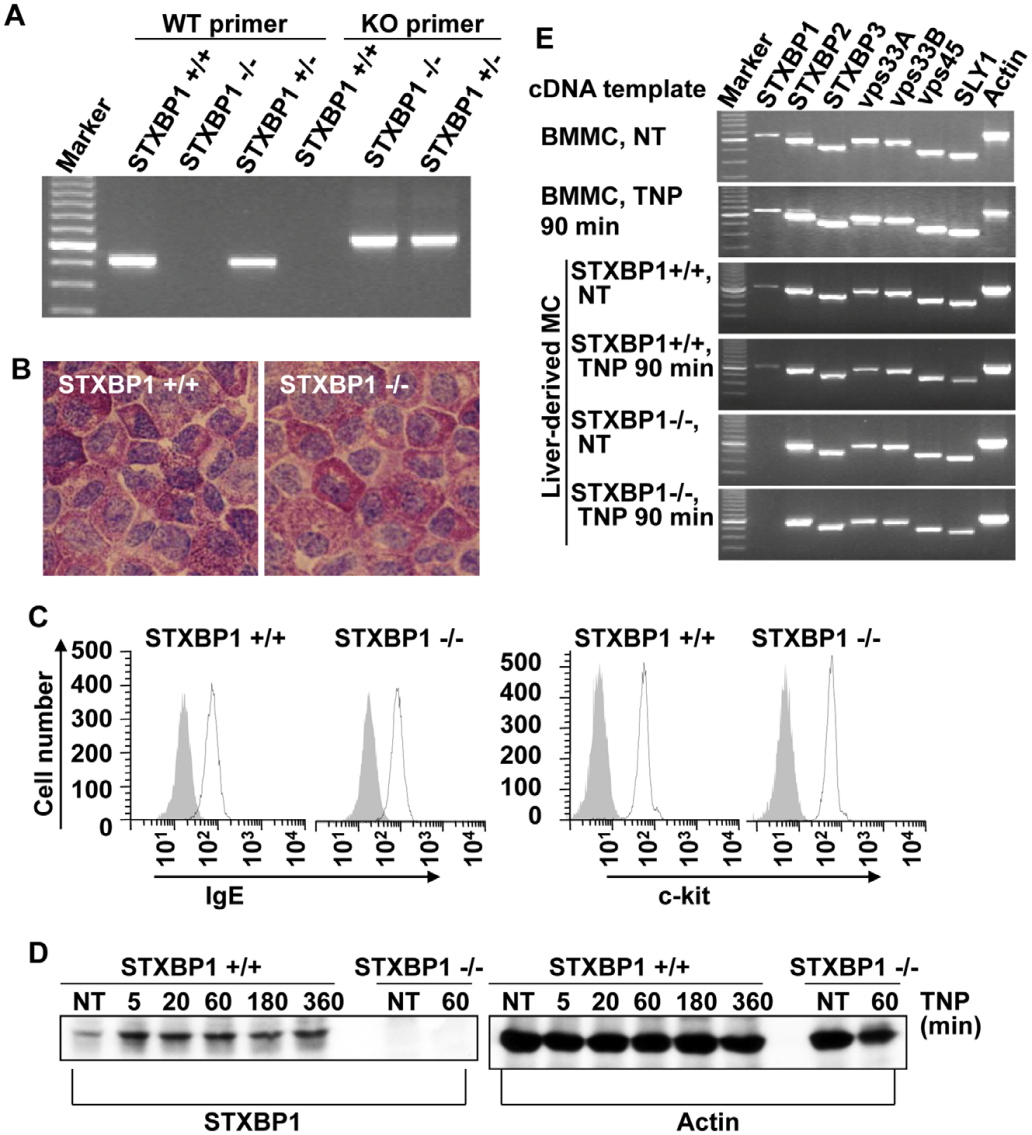
STXBP1 is not required for mast cell maturation. *A,* Wild type, STXBP1 mutant, or heterozygous mice were identified by PCR using DNA from liver cells. *B*, After 4–5 weeks of culture, liver-derived mast cells (LMC) were examined by toluidine blue staining to determine mast cell purity. A representative photomicrograph is shown (original magnification = ×100). *C*, LMCs were sensitized with anti-TNP IgE, stained with FITC-conjugated anti-IgE or anti-c-kit antibodies, and examined by flow cytometry. *D,* STXBP1^+/+^ and STXBP1^−/−^ LMCs were sensitized with anti-TNP IgE and stimulated with TNP-BSA (10 ng/ml) for various times. Whole cell lysates were analyzed by Western blotting for STXBP1 and Actin. *E,* Sec1/Munc18 (SM) family gene expression profiles were identified by RT-PCR using mRNA from wild type mouse bone marrow-derived mast cells (BMMC), and wild type (STXBP1^+/+^) or STXBP1^−/−^ LMCs, with or without TNP-BSA stimulation. All members of SM family are expressed in wild type BMMCs and LMCs. Similar results were observed in 2 independent experiments.

Since *STXBP1*-deficient mice die prematurely due to an absence of neurotransmitter secretion in the brain [39], it is not feasible to use the traditional approach of culturing mast cells from bone marrow cells (BMMC, bone marrow-derived mast cell). Instead, livers from the newborn mice in the STXBP1^+/−^ breeding colony were used to culture mast cells. STXBP1-deficient (STXBP1^−/−^), wild-type (STXBP1^+/+^), or heterozygous (STXBP1^+/−^) mice were identified by PCR-based genotyping (Fig. 1A). Each individual experiment was performed using mast cells derived from the litter matched animals. Liver cells were cultured in SCF and IL-3-containing media for 4–8 weeks and examined by metachromatic staining and flow cytometry. No morphological differences were observed between STXBP1-deficient and wild-type mast cells by toluidine blue staining (Fig. 1B). To further characterize the maturation of STXBP1^−/−^ mast cells, we examined the expression level of major mast cell surface markers. IgE sensitized STXBP1^+/+^ and STXBP1^−/−^ mast cells were stained with antibodies against IgE and c-kit followed by examination by flow cytometry. STXBP1-deficient and wild-type mast cells expressed similar levels of IgE receptor and c-kit (Fig. 1C) suggesting no defect in mast cell maturation in the absence of STXBP1.

To determine STXBP1 expression at the protein level, STXBP1^+/+^ and STXBP1^−/−^ LMC after sensitization with anti-TNP IgE were stimulated TNP-BSA for various times or left untreated (NT). Cells were then lysed and analyzed by Western blot for STXBP1. STXBP1 was detected in STXBP1^+/+^ mast cells, but not in STXBP1^−/−^ mast cells (Fig. 1D). It appears that TNP-BSA stimulation increased the STXBP1 level.

To confirm the absence of *STXBP1* gene expression in STXBP1^−/−^ LMCs, as well as to examine the gene expression level of other sec1/munc18 family members, STXBP1^+/+^ and STXBP1^−/−^ LMCs were sensitized with anti-TNP IgE overnight and stimulated with TNP-BSA for 90 minutes or left untreated (NT). Gene expression was analyzed by RT-PCR using mRNA isolated from these cells. As expected, expression of the STXBP1 gene in STXBP1^−/−^ LMC was not detected, while the expression of other members of the SM family were unimpaired (Fig. 1E). Indeed, all SM family members were expressed in wild-type BMMCs and wild-type LMCs, as previously reported [40].

### STXBP1 does not Impair *in vitro* IgE-dependent Mast Cell Degranulation, Intracellular Calcium Mobilization, or the Production of Reactive Oxygen Intermediates (ROI)

**Figure 2:**
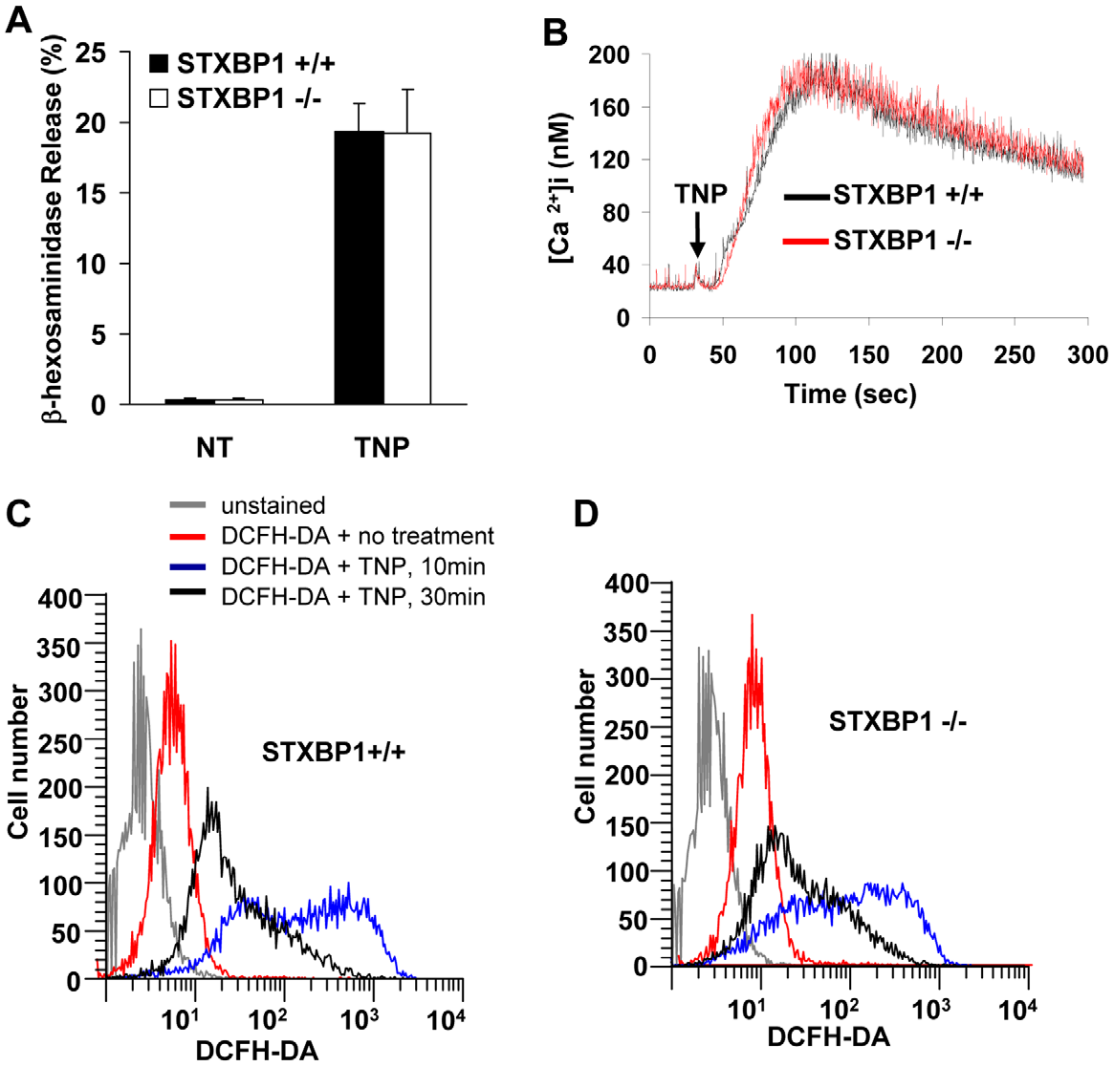
STXBP1 deficiency has no effect on mast cell degranulation, intracellular calcium mobilization, and intracellular reactive oxygen intermediate (ROI) production. *A,* Mast cell degranulation was determined by measuring β-hexosaminidase release after stimulation with TNP-BSA for 20 min. No difference was detectable in mast cell degranulation *in vitro*. (*n* = 4). *B,* Intracellular calcium ion flux analysis of STXBP1^+/+^ and STXBP1^−/−^ mast cells following IgE-mediated mast cell activation. *C* and *D*, Anti-TNP IgE-sensitized STXBP1^+/+^ mast cells (*C*) and STXBP1^−/−^ mast cells (*D*) were incubated with 5 µM dichlorodihydrofluorescein-diacetate (DCFH-DA) for 15 min, and then treated with or without TNP-BSA (10 ng/ml) for various times. The ROI-mediated DCFH-DA oxidation was assayed by flow cytometry. (B-D) The data are representative of three experiments with similar results.

Since STXBP1 plays a key role in eukaryotic trafficking, such as neurotransmitter release in neurons [39], granule release in platelets [22], [41], etc, and since other SM family proteins, such as STXBP2 and STXBP3 are involved in mast cell degranulation [9], we set out to address the role of STXBP1 in mast cell degranulation. To examine whether deficiency of STXBP1 affected mast cell degranulation *in vitro*, STXBP1^+/+^ and STXBP1^−/−^ LMCs were sensitized with anti-TNP IgE and stimulated with TNP-BSA for 20 minutes. The *in vitro* degranulation of LMCs was determined through measurement of β-hexosaminidase release. No impairment of β-hexosaminidase release in STXBP1^−/−^ LMCs compared with WT LMCs was observed (Fig. 2A). Moreover, when histamine release was examined in this *in vitro system*, again no deficiency was detected in STXBP1^−/−^ LMCs compared to wild-type cells (data not shown). Calcium influx is known to maintain and enhance signals after FcεRI-mediated activation. To examine the role of STXBP1 in mast cell calcium mobilization, STXBP1^−/−^ and STXBP1^+/+^ LMCs were sensitized with anti-TNP IgE and preloaded with Fura-2 AM followed by stimulation with TNP-BSA., No impairment in calcium mobilization was observed following FcεRI-mediated activation (Fig. 2B).

Previous studies have demonstrated that intracellular reduction-oxidation reactions participate in mast cell activation leading to mediator release. Increased levels of intracellular reactive oxygen intermediates (ROI) induced through exposure to exogenous agents may enhance or suppress mast cell mediator release [42], [43]. To examine the production of ROIs in STXBP1-deficient LMCs, anti-TNP IgE sensitized STXBP1^+/+^ and STXBP1^−/−^ LMCs were incubated with 5 µM dichlorodihydrofluorescein-diacetate (DCFH-DA) for 15 minutes followed by TNP-BSA stimulation. The ROI-mediated DCFH-DA oxidation was determined by flow cytometry and was found to be unaffected in LMCs derived from STXBP1-deficient mice when compared to wild-type cells (Fig. 2 C &D).

### STXBP1 Deficiency does not Impair Cytokine, Chemokine and PGD2 Production following IgE-dependent Mast Cell Activation

**Figure 3:**
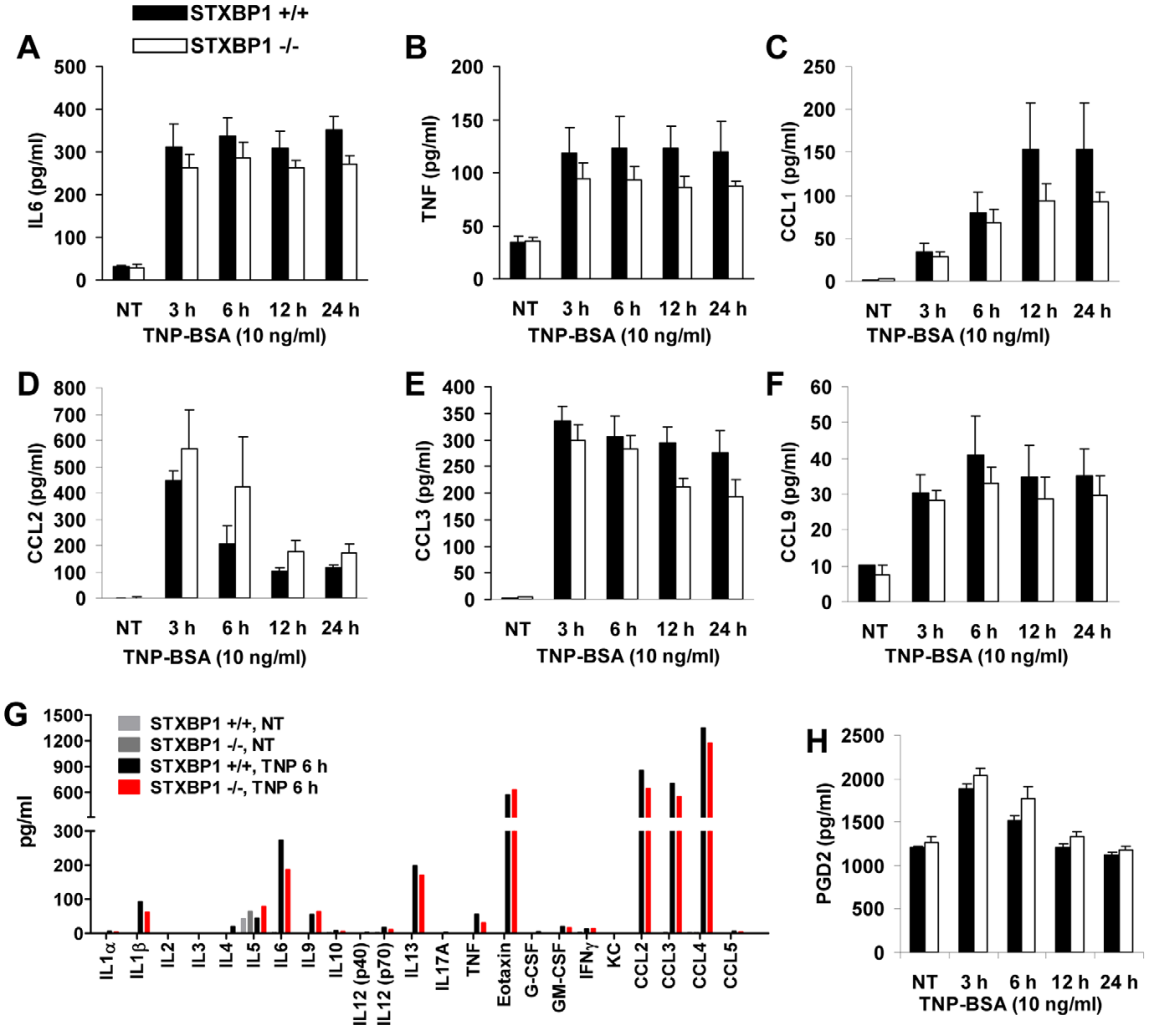
STXBP1 deficiency has no effect on mast cell IgE-dependent cytokine/chemokine and PGD2 production. IL6 (*A*), TNF (*B*), CCL1 (*C*), CCL2 (*D*), CCL3 (*E*), and CCL9 (*F*) production by STXBP1^+/+^ and STXBP1^−/−^ FcεRI-activated mast cells was assessed by ELISA in cell-free culture media following stimulation. (n = 4 separate experiments). *G,* Cytokines and chemokines were detected using a Bio-Plex Pro Mouse 23-plex Group 1 Cytokine Assay (Bio-Rad, Hercules, CA) according to the manufacturer’s protocol. This assay detected 23 cytokines and chemokines including IL-1α, IL-1β, IL-2, IL-3, IL-4, IL-5, IL-6, IL-9, IL-10, IL-12 (p40), IL-12 (p70), IL-13, IL-17A, TNF, Eotaxin, G-CSF, GM-CSF, IFNγ, KC, CCL2, CCL3, CCL4, and CCL5. *H,* PGD2 production by STXBP1^+/+^ and STXBP1^−/−^ FcεRI-activated mast cells was measured using enzyme immunoassay kit. (n = 4 separate experiments).

The activation of mast cells through IgE crosslinking leads to the production of various inflammatory cytokines and chemokines, which play an important role in mast cell-mediated immune responses. To examine the role of STXBP1 in IgE-dependent cytokine and chemokine production by mast cells, LMCs from STXBP1^+/+^ and STXBP1^−/−^ mice were sensitized with anti-TNP IgE and stimulated with TNP-BSA. Cytokine and chemokine production was determined using ELISA in supernatants from activated LMCs. There were slight differences in the levels of TNF, IL-6, CCL1 and CCL2. However, these minor differences did not reach statistical significance so the importance is unclear. All other cytokine and chemokine levels were unchanged (Fig. 3A–F). To examine cytokines and chemokines more broadly, a multiplex protein array simultaneously assessing the production of 23 different mediators was performed on supernatants from wild-type and STXBP1^−/−^ LMCs. Again, no significant impairment in any other pro- or anti-inflammatory cytokines was detected (Fig. 3G) suggesting cytokine secretion is unimpaired in STXBP1-deficient mast cells.

To determine whether STXBP1 affects IgE-mediated lipid mediator production, cell free supernatants from LMCs with or without TNP-BSA stimulation were used to determine PGD2 production. STXBP1^+/+^ and STXBP1^−/−^ mast cells produced similar amounts of PGD2 (Fig. 3H).

### STXBP1 Deficiency does not Affect IgE-dependent IκB-NFκB Pathway Activation, MAP Kinase Phosphorylation, or Transcription Factor Activation

**Figure 4:**
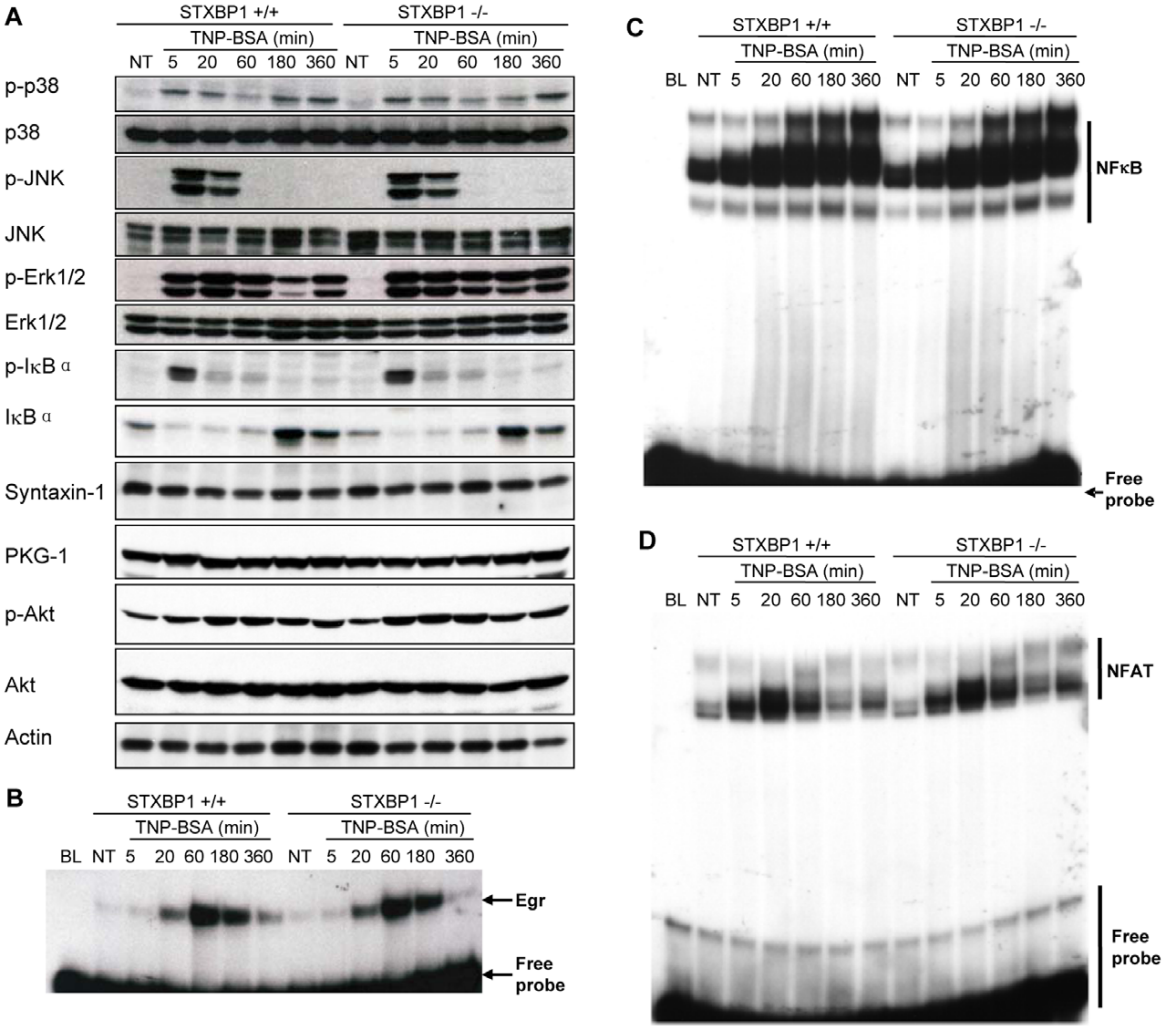
STXBP1 is not required for IκB-NFκB pathway activation, MAP kinase phosphorylation, or Egr and NFAT activity in FcεRI-activated mast cells. *A*, STXBP1^+/+^ and STXBP1^−/−^ mast cells were sensitized with anti-TNP IgE and stimulated with TNP-BSA (10 ng/ml) for various times. Whole cell lysates were analyzed by Western blotting for phospho-IκB and total IκB, phospho-p38 and total p38, phospho-JNK and total JNK, phospho-ERK1/2 and total ERK1/2, phospho-Akt and total Akt, Syntaxin-1, PKG-1, and Actin. *B-D,* STXBP1^+/+^ and STXBP1^−/−^ mast cells were sensitized with anti-TNP IgE and stimulated with TNP-BSA (10 ng/ml) for various times. The nuclear proteins were isolated and subjected to EMSA. Egr binding consensus sequence (5′-GGA TCC AGC GGG GGC GAG CGG GGG CGA ACG-3′) (*B*), NF-κB binding consensus sequence (5′-TTA TCA AAT GTG GGA TTT TCC CAT-3′) from IL-6 promoter (*C*), and NFAT binding concensus sequence (5′-AAG GTG TTT CCC CAA GCC TTT CCC-3′) from IL-13 promoter (*D*) were labeled with ^32^P for EMSA. Shown is a representative from three independent experiments.

Signaling mechanisms regulated by various kinase proteins are thought to contribute to orchestrating intracellular trafficking during mast cell degranulation. STXBP1 itself is a target for phosphorylation that is required for membrane fusion in endothelial cells [44], [45], [46], [47]. To determine whether STXBP1 plays a role in the signaling pathways initiated in mast cells following activation through FcεRI, STXBP1^+/+^ and STXBP1^−/−^ LMCs were sensitized with anti-TNP IgE and stimulated with TNP-BSA. Cell lysates were analyzed by Western blotting to detect phospho- or total IκB, p38, JNK, ERK1/2, Akt, and PKG-1. We did not observe major difference in the expression or phosphorylation of these proteins (Fig. 4A). Syntaxin-1 is a STXBP1 binding partner. Similarly, no major difference of syntaxin-1 was found between STXBP1^+/+^ and STXBP1^−/−^ LMCs (Fig. 4A).

Previous work in our lab has shown that Egr1 plays an important role in regulating mast cell activation and cytokine production [38], [48], [49]. To examine the effect of STXBP1 deficiency on Egr1 transcriptional activity, LMCs from wild-type and STXBP1^−/−^ mice were sensitized using anti-TNP IgE and stimulated with TNP-BSA for various times. Nuclear proteins of these LMCs were isolated and analyzed by EMSA using a ^32^P-labeled Egr family binding consensus sequence as a probe. STXBP1 deficiency showed no effect on Egr1 transcriptional activity (Fig. 4B). The pro-inflammatory transcription factors NFκB and NFAT also play an important role in the regulation of *de novo* synthesis of cytokines and chemokines following IgE-FcεRI stimulation. We examined the effect of STXBP1-deficiency on NFκB (Fig. 4C) and NFAT (Fig. 4D) activity through EMSA. STXBP1 deficiency had no effect on NFκB or NFAT activity. Together, these results suggest that mast cell pro-inflammatory transcription factors are fully active in the absence of STXBP1.

### STXBP1-deficiency does not Impair Passive Cutaneous Anaphylaxis and IgE-mediated Late-Phase Cutaneous Reaction *in vivo*

**Figure 5:**
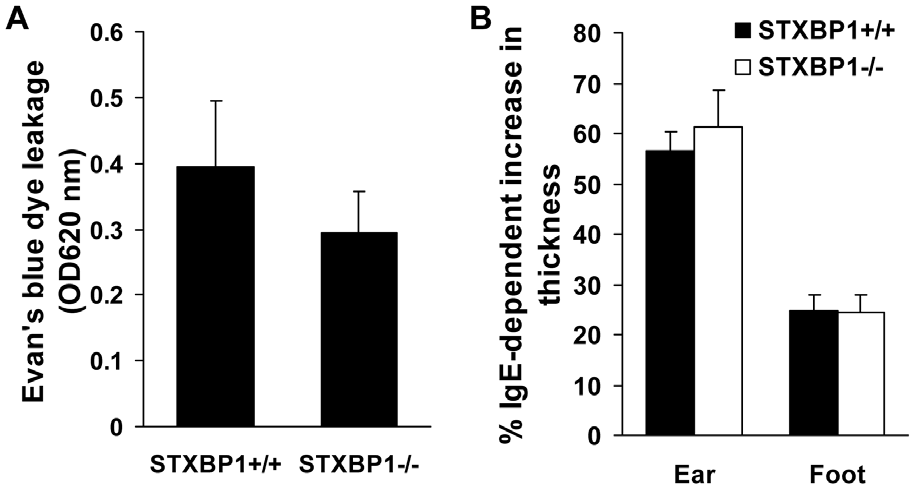
STXBP1 deficiency has no effect on Passive Cutaneous Anaphylaxis (PCA) reactions and late-phase cutaneous anaphylaxis *in vivo*. Mast cell-deficient W^sh^/W^sh^ mice were reconstituted locally in the ears and footpads by intradermal injection of wild-type (left ear and left footpad) and STXBP1^−/−^ (right ear and right footpad) mast cells. *A,* Mast Cell-reconstituted W^sh^ mice or W^sh^ mice (background) were sensitized by intradermal injection of anti-DNP IgE into ears. After 24 hours, mice were challenged by intravenous injection of antigen (DNP-HSA) in 0.5% Evan’s blue. After 30 minutes, Evan’s blue was extracted and the intensity of the dye was measured by absorption at 620 nm. Data are mean ± SEM, *n* = 6. *B,* Mast cell-reconstituted W^sh^ mice were sensitized by intravenous injection of anti-DNP IgE. Twenty-four hours later, a cutaneous reaction was elicited by the application of 20 µl of a solution of 0.3% (weight/volume) dinitrofluorobenzene (DNFB) in acetone/olive oil (4∶1) to the ears and footpads. After another 24 hours, the cutaneous reaction was assessed by comparing the footpad or ear thickness before and after DNFB stimulation according to measurements with a micrometer. Data are mean ± SEM, *n* = 6.

Mast cell mediators released by degranulation contribute to the anaphylactic reaction *in vivo*. To examine whether the deficiency of STXBP1 has any effect on this pathological process, mast cell-deficient (W^sh^) mice were reconstituted with STXBP1^+/+^ and STXBP1^−/−^ LMCs locally at the ear and footpad via intradermal injection. Eight weeks later, the LMC-reconstituted W^sh^ mice were then sensitized by anti-DNP IgE via intradermal injection. Twenty four hours later, mice were challenged systemically with DNP-BSA in Evan’s blue dye via tail vein injection. Thirty minutes later, tissue from both ears were collected for extraction of Evans blue dye to determine vascular permeability. No effect on vascular permeability was observed in STXBP1-deficient LMC-reconstituted W^sh^ mice when compared to wild-type reconstituted controls (Fig. 5A).

Activated mast cells are involved in the development of late-phase allergic responses [45], [46]. To examine whether STXBP1 contributes to FcεRI-mediated late-phase cutaneous reaction *in vivo*, STXBP1^+/+^ or STXBP1^−/−^ LMC-reconstituted W^sh^ mice were sensitized with anti-DNP IgE 24 hours before a solution of 0.3% dinitrofluorobenzene (DNFB; hapten) was applied epicutaneously to the ear skin or footpad. IgE/antigen-specific edema was determined by tissue thickness. STXBP1^−/−^ LMC-reconstituted W^sh^ mice again showed no defect in IgE/antigen-specific edema as determined by tissue thickness, when compared to wild-type reconstituted controls (Fig. 5B), indicating that STXBP1-deficiency does not impair allergic reactions in mice.

## Discussion

The current study was designed to understand the role of STXBP1 in IgE-dependent mast cell activation including mast cell degranulation, IgE-dependant signaling pathway activation, and cytokine/chemokine production. One of major effector functions of mast cells is the release of preformed granules immediately following stimulation. Like other vesicular trafficking steps, mast cell degranulation is mediated by complex pathways. It has been shown in previous studies that the binding of STXBP1 to SNARE proteins is sufficient to prime membrane fusion [50]. It has also been shown that deletion of STXBP1 abolishes exocytosis at neuronal synapses [39] and in platelets [22], [41], and other SM family proteins, such as STXBP2 and STXBP3 are involved in mast cell degranulation [9]. Given the importance of STXBP1 in the interaction with SNARE complexes, one might expect that mast cell degranulation would be impaired in the absence of STXBP1. However, in the current study we did not observe any impairment of mast cell degranulation, suggesting that the mast cell degranulation process might be regulated by SM family members other than STXBP1. A redundant system of STXBPs in mast cells, permitting normal exocytosis in mast cells in the absence of STXBP1, seems likely. A possible mechanism for such redundancy could be STXBP2 (Munc18-2) which was shown to be overexpressed in mast cells and regulate mast cell degranulation by binding to SYNTAX 2/SNARE23 [40].

Another major effector mechanism employed by mast cells is to activate a series of signaling cascades, which lead to the production of various cytokines and chemokines, and regulate the innate and adaptive immune responses [1]. Previous studies have demonstrated that phosphorylation of STXBP1 in neuronal cells [44] and STXBP3 and syntaxin 4 in endothelial cells [45] promotes STXBP-syntaxin dissociation, thereby facilitating vesicular fusion. Phosphorylation of syntaxins, STXBP and SNAP-23 is catalyzed by kinases such as protein kinase C (PKC) [45], [46]. Additionally, cGMP-dependent protein kinase (PKG) and PI3K are involved in mast cell degranulation with potential roles in phosphorylation of target membrane SNARE complex proteins [47]. Altogether this suggests that SNARE complex proteins may be involved in intracellular signaling mechanisms. In our study, we examined the activation of various signaling pathways including MAP kinases JNK, p38, Erk1/2, Akt (PI3K), PKG, IκB-NFκB, as well as NFAT, that are activated following the cross-linking of FcεRI. However, we did not find any impairment in any of these pathways in STXBP1^−/−^ mast cells. Our previous data revealed a regulatory role for the transcriptional factor Egr1 in mast cell activation [38], [48]. However we did not observe any difference in Egr1 transcriptional activity between STXBP1^+/+^ and STXBP1^−/−^ mast cells. Moreover, STXBP1-deficient mast cells showed no major impairment in cytokine and chemokine production. Reconstitution of STXBP1^+/+^ and STXBP1^−/−^ mast cells into W^sh^ mice showed a similar results that STXBP1 deficiency has no effect on IgE-medated allergic reaction. Our data suggest that although STXBP1 together with all other members of SM family are expressed in BMMCs and LMCs, STXBP1 deficiency has no effect on mast cell maturation and IgE-dependent mast cell activation *in viro* and *in vivo*. These findings suggest either functional redundancy in STXBPs in mast cells, or that an unknown function for STXBP1 in mast cells may yet exist.
